# Associations of dinner-to-bed time, post-dinner walk and sleep duration with colorectal cancer: A case-control study

**DOI:** 10.1097/MD.0000000000012038

**Published:** 2018-08-24

**Authors:** Yanjuan Lin, Yanchun Peng, Bing Liang, Shenshan Zhu, Lin Li, Fei Jang, Xizhen Huang, Yuhong Xie

**Affiliations:** a Department of Nursing; b Department of Colorectal surgery, Fujian Medical University Union Hospital, Fujian Province, China.

**Keywords:** colorectal cancer, dinner-to-bed time, post-dinner walk, sleep duration

## Abstract

Colorectal cancer (CRC) ranked 3rd for cancer incidence and 4th for cancer death worldwide. Despite the increasing number of CRC studies, the etiology is not yet clear. In this study, we investigated the effects of the dinner-to-bed time, post-dinner walk and sleep duration on the risk for CRC.

We conducted a matched case-control study based on hospital population. We involved 166 patients had a newly histologically confirmed CRC without previous treatment and 166 healthy healthy residents matched by age and gender at Fujian Medical Union Hospital. A self-designed questionnaire was used to information on demographic characteristics, dinner-to-bed time, post-dinner walk, sleep duration, and other behavioral factors. Conditional logistic regression was used to calculate the odds ratio (OR) and 95% confidence intervals (95% CIs) to assess the effect of dinner-to-bed time, post-dinner walking, and sleep duration as well as their joint effect on the risk of CRC at different genders.

The adjusted odds ratio (AOR) of CRC for subjects with shorter dinner-to-bed time (2.0–2.9 h) were 2.527 (95% CIs = 1.127–5.337), relative to those with longer dinner-to-bed time (≥4 h), the difference was statistically significant (*P* < .05). Post-dinner walk was associated with a significantly decreased CRC risk (AOR = 0.339, 95% CIs = 0.203–0.865) compared with post-dinner non-walk. Compared with 6–9 h of sleep duration, the risk OR of CRC were 3.843 (95% CIs = 2.767–7.800, *P* < .05) and 2.12 (95% CIs = 0.754–5.959, *P* > .05) for long (≥9 h) and short (<6 h) sleep duration. The risk of CRC individuals with shorter dinner-to-bed time and post-dinner non-walk caused higher risk than those with longer dinner-to-bed time and post-dinner walk (AOR = 3.361, 95% CIs = 2.043–6.316). The risk of CRC was 2.231 (95% CIs = 1.089–3.762, *P* < .001), with a shorter dinner-to-bed time and ≥9 hours of sleep duration.

We found that shorter dinner-to-bed time (<3 h), post-dinner walk, and long sleep duration (≥9 h) were seems to be related to CRC and may increase the risk of CRC.

## 1 Introduction

The global burden of cancer data in 2015 showed that the incidence rate of Colorectal cancer (CRC) morbidity accounted for 3rd and 4th cancer deaths worldwide, with 1,573,000 new cases and 771,000 deaths, all of these deaths occur in developing countries, with more than two-thirds.^[1]^ About 22% of the world's new cancer cases and 27% of cancer deaths occur in China.^[2]^ In China, the incidence of CRC accounted for 5th, and the mortality rate accounted for 5th, of which 3,763,000 of CRC new case, accounting for 88% of the proportion of cancer, and 191,000 death, accounting for 6.8%.^[3]^

There are a variety of screening methods for CRC, and endoscopy is the main means of early diagnosis. However, China has a large population base and limited medical resources, so endoscopy can not be used as a common screening method. Therefore, it is vital to identify the risk factors associated with CRC to implement effective preventions and control measures. The development of CRC is widely considered to be a multifactorial and complex process. So far, the exact etiology of CRC is still unclear. However, a number of studies have shown that the adverse lifestyle is the main cause of CRC.^[4,5]^ In 2005, Yasuhiro Fujiwara et al,^[6]^ a Japanese scholar, first proposed the concept of the dinner-to-bed time, which is the usual time interval from dinner finished to going to bed. Some studies have showed that dinner-to-bed time was associated with the occurrence of esophageal squamous cell carcinoma (ESCC), gastric cardia adenocarcinoma (GCA), and gastric cancer (GC),^[7–9]^ but the effect of dinner-to-bed time on the development of CRC has not been studied. Ratjen et al's^[10]^ prospective study of 1376 CRC patients found that physical activity (including walking, cycling, sports, and gardening) could reduced the mortality risk in cancer survivors, prevent cancer recurrence and prolong life in cancer survivors. But there is no study of the effect of post-dinner walk on CRC. In addition, sleep duration is associated with CRC, but the findings are controversial.

At present, there is no research on the relationship between dinner-to-bed time, post-dinner walk, and sleep duration with the risk of CRC. Therefore, we conducted a population-based case-control study to assess the effects of dinner-to-bed time, post-dinner walks, and sleep duration on CRC.

## 2 Materials and methods

### 2.1 Study subjects

The study was a population-based, case-control study in Fujian Medical University Union Hospital during June 2017 to January 2018. A total of 332 patients were invited to participate in the study. There were 166 patients newly diagnosed with CRC in the case group. The case group inclusion criteria: 18 to 75 years old; newly diagnosed primary incident CRC cases with endoscopic biopsy or pathological histologic confirmation; no previous history of a malignant tumor; no long-term history of chronic disease (diabetes); not with other serious physical illness; and voluntary participation. The case group exclusion criteria: patients with a history of mental illness and mental retardation. The control group randomly selected the same period of time with patients age and gender matched physical examination healthy persons, 166 healthy people were selected as the control group from Fujian Medical University Union Hospital. The control group inclusion criteria: matched with case group, age, and gender; no previous history of a malignant tumor; no previous surgery related to the digestive system; no previous surgery related to the digestive system; no long-term history of chronic disease (diabetes); not with other serious physical illness; and voluntary participation. The control group exclusion criteria: patients with a history of mental illness and mental retardation.

This study was approved by Fujian Medical University Union Hospital's ethics Review Committee. All participants provided written consent for their information to be collected and used for research, and they had the right to withdraw from the study at any time without prejudice.

### 2.2 Data collection

Through uniform training of undergraduate and above nursing staff, self-designed questionnaires were used to conduct face-to-face surveys of subjects meeting the research criteria. Patients who were unable to communicate properly due to illness were investigated for their close relatives who lived together. Patients were interviewed at the hospital within 1 week of receiving a diagnosis of CRC, and control group was interviewed in medical examination center during the same period. All participants provided information about their past 15 years before undiagnosed CRC, including 3 parts. The 1st part was general demographic characteristics including age, gender, height, weight, education level, marital status, occupation, living status, and local environmental pollution. The 2nd part was personal habits including dinner-to-bed time, post-dinner walk, sleep duration, smoking, and drinking (Researchers asked participants: what time do you usually have dinner? Do you take a walk outdoors after dinner? If the answer is yes, then asked how long each time do you take a walk? If the time exceeds 30  min, then continue to ask how many times a week walk? What time do you go to bed at night? If the answer is yes, the researchers asked participants: do you wake up often? If the answer is more than 3 times per week, the researchers continue to asked participants: how long do you take to fall asleep again? What time do you get up in the morning? Do you smoke? Do you drink?). The last part is dietary habit including fresh vegetable intake, fresh fruit intake and special dietary habit, and family history of cancer (How many vegetables do you eat for a week? How many times a week do you eat fruit? Do you eat pickled foods? If the answer is yes, then ask how many times a week you eat it? Whether to eat spicy, mildewed, smoked, or fried foods ask the method is the same as pickled foods).

### 2.3 Variables and definitions

Dinner-to-bed time was defined as the usual time interval from dinner finished to going to bed.^[7]^ Post-dinner walk habits were defined as participants reporting, on a weekly basis, taking a walk indoors or outdoors after supper for at least half an hour at a time, and for at least half the number of weeks every year in the past decade.^[8]^ Sleep duration is based on the question “how many hours of sleep do you get per night?” Body mass index (BMI) is weight (kg) divided by height (m) squared (body height and weight 5 years before interviewed).^[11]^ Smoking was defined as more than 1 cigarette per day for at least 6 months.^[12]^ Alcohol drinkers were defined as those who reported consuming at least 1 drink per week (beer 500 mL, wine/liquor 200 g) for more than half a year.^[13]^ Special dietary habits included eating pickled, spicy, mildewed, smoked, or fried foods on a daily basis.^[7]^ A family history of cancer means the 1st-degree relatives with any type of cancer.^[14]^ Local environmental pollution was assessed as living in a residential area within 5 km of an environmental hazard, such as polluted air or water, etc.^[15]^

These variables we collected were categorized as follows: dinner-to-bed time (shorter: <3 h; moderate: 3–3.9 h; longer: ≥4 h), post-dinner walk (walk or non-walk), sleep duration (shorter: <6 h; moderate: 6–9 h; longer: ≥9 h).

### 2.4 Quality control

The study quality control included:

(1) (1)The design of the questionnaire was based on literature, epidemiologists, and CRC experts.
(2) A double-blind research design with regard to both the research hypothesis and objectives.
(3) A unified standard was used to train investigators and train qualified individuals to begin investigations.
(4) The investigation was conducted in a quiet environment using plain language.
(5) All participants answered the questions personally and each interview took approximately 20 minutes to complete.
(6) After the survey, the researchers checked the integrity and validity of the questionnaire on the spot and immediately completed the missing items. At the same time, we took a random check on the completion of the questionnaire to avoid the invalid questionnaires. To ensure effective response rate.

### 2.5 Statistical analysis

The database was established using Epidata 3.1 (via double entry). The original data were checked logically and checked by random sampling 10%. The data were analyzed using the SPSS statistical package, version 21.0. Categorical variables and numerical variables in patients and controls were compared by Chi-square test and Student's *t* test. The conditional logical regression model was used to calculate the odds ratio (OR) and 95% confidence intervals (CIs) to evaluate the correlation and risk between the variables and the CRC, after being matched by age and gender. The multivariable logistic regression model was used to calculate the adjusted odds ratio (AOR), to measure the relationship between the related factors and the corresponding cancer intensity. All *P* values were 2 sided and a *P* value <.05 was statistically significant.

## 3 Results

A total of 217 patients met the inclusion criteria of the case group, but only 166 agreed to participate in our study. The total sample size of the study was 332, of which the case group and the control group were 166 cases. The baseline characteristics of study subjects are summarized in Table 1. The mean age was 57.50 ± 10.72 years old and 55.58 ± 9.68 years old *t*=1.715, *P*=.087. The average BMI was 23.08 ± 2.90 kg/m^2^ and 22.93 ± 2.41 kg/m^2^*t*=0.52, *P* = .600. There was no statistically significant in age, gender, BMI, education level, marital status, occupation, living area, and local environmental pollution between the 2 groups. The percentages of smokers, family cancer history, and special dietary habits were all higher in CRC patients than in controls. The dinner-to-bed time in case group was significantly shorter than that in the control group (*P* < .05), and the proportion of post-dinner walk was lower than that in the control group (*P* < .001). The difference between the 2 groups at sleep duration was statistically significant (*P* < .001).

**Table 1:**
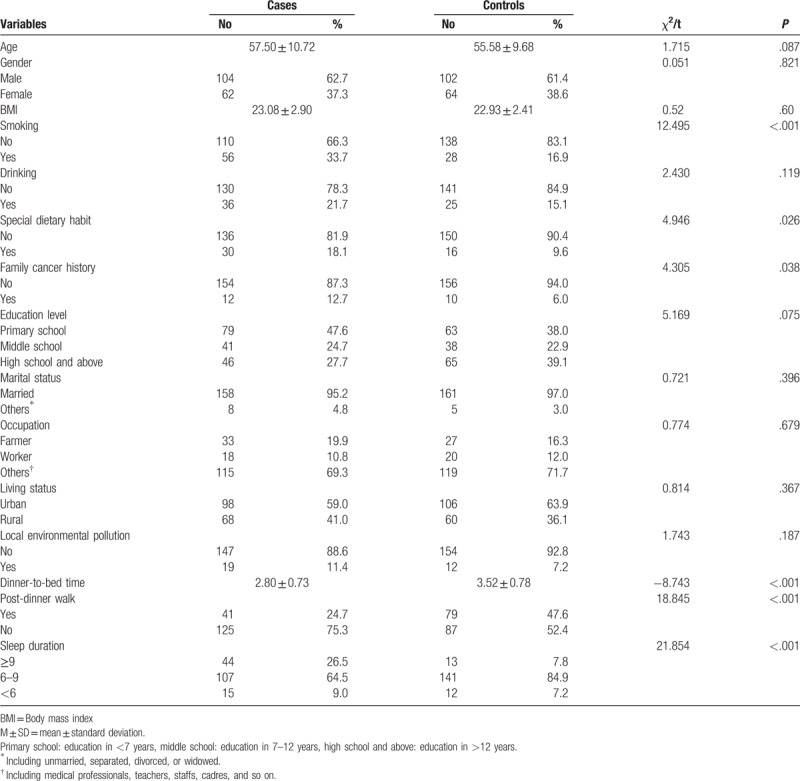
Characteristics of colorectal cancer cases and controls.

The risks of CRC in connection with dinner-to-bed time, post-dinner walk, and other factors are shown in Table 2. In univariable analysis, the AORs of CRC for subjects with shorter dinner-to-bed time (2.0–2.9 h) were 2.864 (95% CIs = 1.016–5.069), relative to those with longer dinner-to-bed time (≥4 h). Post-dinner walk was associated with a decreased risk of CRC (AOR = 0.339, 95% CI = 0.203–0.865). The sleep duration of 6 to 9 hours was set as the reference group, the results showed that sleep duration was ≥9 h (AOR = 4.492, 95% CIs = 2.304–8.757) and < 6 h (AOR = 2.708, 95% CIs = 1.017–7.209) increased the risk of CRC. In addition, smoking, special dietary habits, fresh vegetables, fresh fruit, and the family history of cancer were associated with CRC.

**Table 2:**
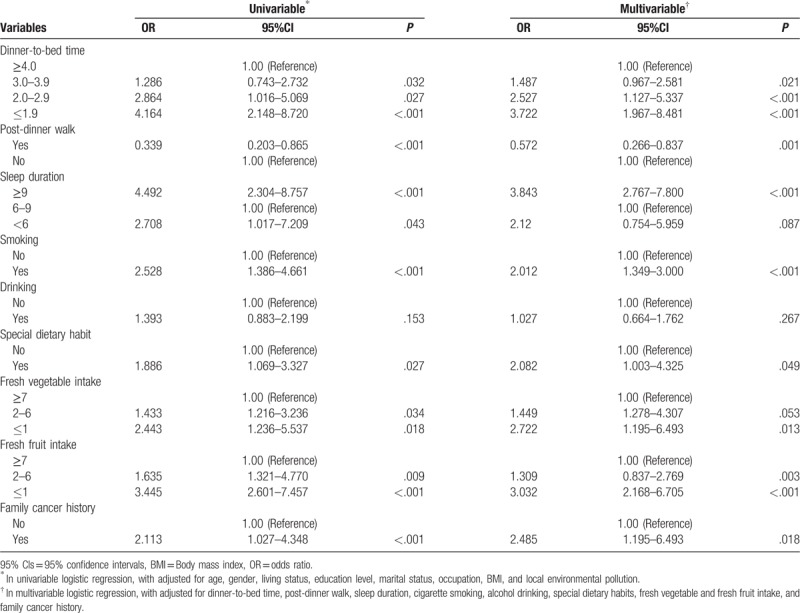
OR (95% CIs) for the association of colorectal cancer risk among dinner-to-bed time, post-dinner walk, sleep duration, and other behavior-related factors.

We further analyzed the impact of dinner-to-bed time on the risk of CRC in the model stratified according to post-dinner walk habits and sleep duration (Table 3). Shorter dinner-to-bed time in the post-dinner non-walk group (AOR = 3.361, 95%CIs = 2.043–6.316, *P* < .001) compared to the post-dinner walk group (AOR = 2.175, 95% CIs = 1.790–5.589, *P* < .013), caused a higher risk of CRC. Compared with the 6 to 9 hours sleep duration group, AOR was 2.231 (95%CIs = 1.089–3.762, *P* < .001) for longer sleep duration (95% CIs = 1.089–3.762, *P* < .001), and the shorter sleep duration (<6 h) was 1.634 (95% CIs = 0.766–2.832, *P* = .366).

**Table 3:**
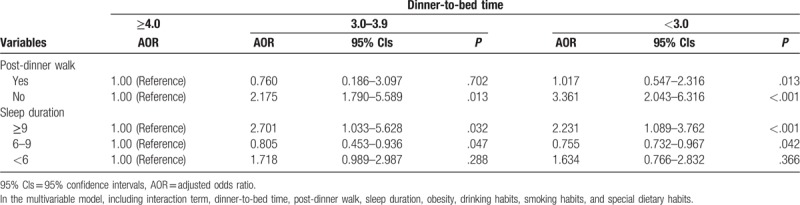
Adjusted odds ratios of colorectal cancer for dinner-to-bed time according to a post-dinner walk and sleep duration.

Table 4 showed the effect of dinner-to-bed time, post-dinner walk, and sleep duration on CRC in different genders. After adjusting general demographic data, smoking, drinking, special dietary habits, family history of cancer, environmental pollution in living areas and other variables, the results showed that shorter dinner-to-bed time increased the risk of CRC in male, and the shorter the interval, the higher the risk of CRC (*t* = 2–2.9 h: AOR = 1.306, 95% CIs = 1.079–3.509, *P* = .048; *t*≤1.9 h: AOR = 3.338, 95% CIs = 1.575–6.707, *P* = .029). Shorter dinner-to-bed time in females (*t*≤1.9) increased the risk of CRC (AOR = 2.703, 95% CIs = 1.102–4.842, *P* = .031). Post-dinner walk showed a considerably stronger effect on the risk for CRC than post-dinner non-walk; in the males the AOR was 0.437 (95% CIs = 0.229–0.937, *P* = .012), in the females the AOR was 0.180 (95% CIs = 0.07–0.462, *P* < .001). In the subjects with sleep duration of ≥9 hours, the risk of CRC in males was 2.03695 (95% CIs = 1.963–4.302, *P* < .0362), the risk of CRC in females was 1.557 (95% CIs = 1.292–4.487, *P* < .035). At a sleep duration of < 6 hours, the risk of CRC in males was 1.323 (95% CIs = 1.091–2.891, *P* = .038), but there was no significant difference in females (*P* = .789). (Table 4)

**Table 4:**
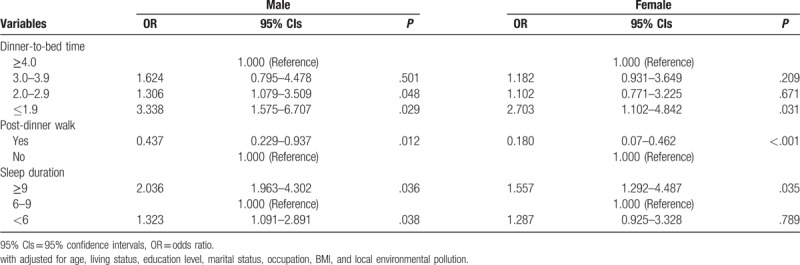
Stratified by gender analysis.

## 4 Discussion

In epidemiological studies, many scholars have suggested that red and higher intake of processed meats, lack of physical activity, overweight and obesity, smoking, and alcohol consumption are considered risk factors for CRC. However, there is no research about the relationship between dinner-to-bed time, post-dinner walk, and sleep duration, and the relationship is analyzed and discussed in this study.

Although scholars have conducted extensive research on the etiology of CRC, it is still unclear. Japanese scholar Yasuhiro Fujiwara et al^[6]^ first found that dinner-to-bed time of ≤2.9 hours was the risk factor for gastroesophageal reflux disease. The effects of dinner-to-bed time and post-dinner walk on the tumors were only found in ESCC and GC. This study found that the average dinner-to-bed time was shorter in the case group than in the control group; dinner-to-bed time of <3 hours was a potential independent risk factor for CRC and may increase the risk of CRC. Shorter dinner-to-bed time may increase the incidence of CRC, considering the following possible mechanisms. The increase of insulin-like growth factor-I (IGF-I) can lead to the occurrence of CRC. It has been observed that night-diet-induced thermogenesis (DIT) is significantly lower than morning^[16]^ and afternoon; moreover, the lipid oxidation at evening was lower than in the morning.^[17]^ Study showed that glucose tolerance impairs in the afternoon and evening, and insulin sensitivity decreased at night, glucose is lower in the afternoon and evening than in the morning, both hyperglycemia and hyperinsulinemia can stimulate increased production of the mitogenic protein IGF-I.^[18]^ In the colorectal, IGF-I halts apoptosis and promotes cell proliferation and tumor growth.^[19]^ Studies have shown that obesity increases the risk of CRC. Over time, there has been a reduction in daytime energy intake with a commensurate increase in mid-afternoon and evening intakes.^[20]^ As foods and beverages consumed in the evening tend to be more energy dense,^[21]^ dinner is typically the most energy-dense meal of the day, which leads to an increase in body weight. Obesity could promote in CRC by changing the metabolic state of the body, releasing a large amount of inflammatory factors, causing intestinal microecologic maladjustment and immune system damage.^[22]^ In addition, when the dinner is too full, protein foods cannot be completely digested, and toxic substances are produced under the action of intestinal bacteria. Furthermore less activity and the intestinal wall peristalsis slowly during sleep prolonged the duration of the toxic substances in the intestinal tract, and increase the incidence of intestinal cancer.

It's still remain controversial that the impact of physical activity on the risk of CRC.^[23]^ Studies have showed that physical activity reduced the risk of CRC-colon adenoma by 15% and reduces the risk of advanced adenomas by 35%,^[24,25]^ and the most common form of physical activity is walking.^[23,26]^ Song et al^[7]^ showed that post-dinner walk was associated with a decreased risk of ESCC (adjusted OR = 0.64, 95% CIs = 0.41–0.89). Xu Le et al^[9]^ found that dinner-to-bed time <3 hours and post-dinner non-walk are independent risk factors for GC. This study found that the risk of developing CRC in shorter dinner-to-bed time and post-dinner walk (OR = 1.017, *P* = 0.013) was lower than that of shorter dinner-to-bed time and post-dinner non-walk (OR = 3.361, *P*<.001), suggesting that post-dinner walk may weaken the risk of a shorter dinner-to-bed time on the CRC. Post-dinner walk, as a protective factor for CRC, there may be the following mechanisms. Regular physical activities not only reduce the concentration of inflammatory factors, but also increase anti-inflammatory cytokines, enhance the body's resistance, and reduce the incidence of cancer.^[27]^ Meanwhile physical activities can reduce the exposure of mucosa to carcinogens in diet after exercise, decrease body fat and insulin resistance, improve immune function, as well as changes in prostaglandin synthesis, modifications in gallbladder acids and cholesterol level as well as gastrointestinal hormones, which can explain the correlation between CRC risk and physical activity.^[28]^ In addition, the study confirmed that physical activity inhibited the growth of tumor cells by lowering the level of IGF-1 in the blood and IGF-1 levels in cancer patients with active physical activity are significantly reduced, and the quality of life of patients is significantly improved.^[29]^

Both decreased sleep duration and increased sleep duration have been associated with CRC in recent studies. Tompson and colleagues studied 1240 patients and found that those with <6 hours of sleep per night had close to a 50% increased risk of colorectal adenomas compared with patients who slept 7 hours or more.^[30]^ Zhang et al^[31]^ found that compared with slept 7 hours per night, slept ≥9 hours per night had an increased risk of CRC in males, it was not found that sleep duration <6 hours was associated with risk of CRC. However, the results of this study showed that the risk of CRC increased in men with a sleep duration of ≥9 hours and a sleep duration of <6 hours. Susan et al^[32]^ followed American females for approximately 6 years and found that the risk of CRC was increased in those with insufficient sleep duration (3–6 h) or longer (≥10 h) compared to sleep duration of 7–9 hours. However, our study only found that the risk of CRC was increased in women with ≥9 hours sleep duration (OR = 1.557, *P* = .035), and there was no statistically significant difference between sleep duration of <6 hours and CRC (OR = 1.287, *P* = .789), indicating that sleep duration of <6 hours does not increase the risk of CRC. There have been many proposed mechanisms for the potential increased risk. Release of inflammatory cytokines in patients with sleep changes can potentially set the stage for the development of CRC. A longer sleep duration leads to increased proinflammatory cytokine release, specifically of interleukin-1 (IL-1) and tumor necrosis factor (TNF), which play a role in new tumor growth.^[31]^ The shorter sleep duration also increased the level of the inflammatory factor and increased risk of CRC, compared with the 7 hours, and the TNF-a level increased by 8%.^[33]^ Moreover, having a longer sleep duration increases cortisol secretion along with insulin resistance, resulting in obesity; short sleep time can also lead to obesity, obesity is an independent risk factor for CRC.

In addition, the effect of dinner-to-bed time on the risk of CRC was analyzed based on the post-dinner walk and sleep duration. The AOR for post-dinner walk of CRC <3 hours was higher than for non-walk, relative to dinner-sleep time of ≥4 hours. When the subjects were analyzed according to the sleep duration, compared with the longer dinner-to-bed time, the risk of CRC with shorter sleep duration ≥9 hours and sleep duration <6 hours was higher than that of 6 to 9 hours. However, the exact mechanism of how the dinner-to-bed time, post-dinner walk and sleep duration contribute to CRC is till unknown.

## 5 Conclusions

We found that shorter dinner-to-bed time (<3 h), post-dinner walk, and long sleep duration (≥9 h) were seems to be related to CRC and may increase the risk of CRC. Prolonging dinner-to-bed time, regular walking after dinner, and moderate sleep duration may have a positive effect on the prevention of CRC.

In this cross-sectional study, whether there is an association between dinner-to-bed time and the risk of CRC needs further study to confirm. And there are the following limitations: 1st, the choice of the study subject was limited to 1 hospital, which needs to expand the scope of investigation and increase the sample size. Second, as with all retrospective case-control studies, this study had a bias of recall and selection. Third, the control group were healthy residents visiting the same hospital for annual health check-ups, so they may have better health consciousness and habits than case group. Finally, we did not include several other variables, such as the use of drugs (anti-inflammatory drugs), working hours, and diet structure; these factors should be included in future research. The present study is apparently the first to provide evidence that a shorter dinner-to-bed time is associated with CRC. The case group was investigated within 1 week of the disease diagnosis and clearly defined the items in the questionnaire to the recollection bias. The inclusion of recognized risk or protective factors for CRC may reduce the potential confounding factors on the results of the study.

## Acknowledgments

The authors thank the Director Sheng Yang of the Department of Oncology and the Director Pan Chi of the Department of Colorectal Surgery at the Fujian Medical University Union Hospital.

## Author contributions

**Data curation:** Yanchun Peng, Fei Jiang.

**Investigation:** Bing Liang, Shenshan Zhu, Yuhong Xie.

**Methodology:** Yanjuan Lin, Yanchun Peng.

**Supervision:** Xizhen Huang.

**Writing – original draft:** Yanjuan Lin, Yanchun Peng.

**Writing – review & editing:** Yanjuan Lin, Lin Li.
